# GLP-1 and Its Analogs: Does Sex Matter?

**DOI:** 10.1210/endocr/bqae165

**Published:** 2024-12-23

**Authors:** Stina Börchers, Karolina P Skibicka

**Affiliations:** Department of Physiology/Metabolic Physiology, Institute of Neuroscience and Physiology, The Sahlgrenska Academy at the University of Gothenburg, 41390 Gothenburg, Sweden; Department of Physiology/Metabolic Physiology, Institute of Neuroscience and Physiology, The Sahlgrenska Academy at the University of Gothenburg, 41390 Gothenburg, Sweden; Nutritional Sciences Department, The Pennsylvania State University, University Park, PA 16803, USA; Huck Institutes of Life Science, The Pennsylvania State University, University Park, PA 16803, USA

**Keywords:** sex differences, GLP-1, exendin-4, semaglutide, estrogens, obesity

## Abstract

While obesity and diabetes are prevalent in both men and women, some aspects of these diseases differ by sex. A new blockbuster class of therapeutics, glucagon-like peptide 1 (GLP-1) analogs (eg, semaglutide), shows promise at curbing both diseases. This review addresses the topic of sex differences in the endogenous and therapeutic actions of GLP-1 and its analogs. Work on sex differences in human studies and animal research is reviewed. Preclinical data on the mechanisms of potential sex differences in the endogenous GLP-1 system as well as the therapeutic effect of GLP-1 analogs, focusing on the effects of the drugs on the brain and behavior relating to appetite and metabolism, are highlighted. Moreover, recent clinical evidence of sex differences in the therapeutic effects of GLP-1 analogs in obesity, diabetes, and cardiovascular disease are discussed. Lastly, we review evidence for the role of GLP-1 analogs in mood and reproductive function, with particular attention to sex differences. Overall, while we did not find evidence for many qualitative sex differences in the therapeutic effect of clinically approved GLP-1 analogs, a growing body of literature highlights quantitative sex differences in the response to GLP-1 and its analogs as well as an interaction of these therapeutics with estrogens. What also clearly emerges is the paucity of data in female animal models or women in very basic aspects of the science of GLP-1—gaps that should be urgently mended, given the growing popularity of these medications, especially in women.

Despite ample evidence for sex differences in the action of key metabolic hormones and established actions of gonadal steroids on ingestive and motivated behaviors, preclinical studies still do not consistently test female subjects, and when they do, they often fail to show sex-disaggregated data. Similarly, while clinical trials in the areas of obesity or diabetes are generally mandated to include both sexes, they sometimes fail to show sex-specific data or appropriately analyze sex differences. Likewise, safety and pharmacokinetics studies, including those evaluating glucagon-like peptide-1 (GLP-1) analogs still tend to exclude female subjects. Notwithstanding these omissions, a growing body of preclinical and clinical literature showcases potential sex differences in the actions of GLP-1 and its analogs.

Here we review the existing preclinical evidence for sex differences, initially focusing on the interaction of sex and sex steroids with food intake and metabolic effects of GLP-1 (Fig 1). We next discuss available clinical evidence (Fig. 2). Lastly, we evaluate available evidence for sex as a factor modulating effects of GLP-1 analogs on emotionality, eating disorders, cardiovascular disease, and the reproductive system. These questions are of vital importance both for an accurate understanding of the basic science of GLP-1 and for the clinic, especially in light of recent data indicating that use of GLP-1-based therapeutics is spiking among young women more than men, with women representing over 75% of young (18-25 years old) patients taking GLP-1 analogs (1).

**Figure 1.**
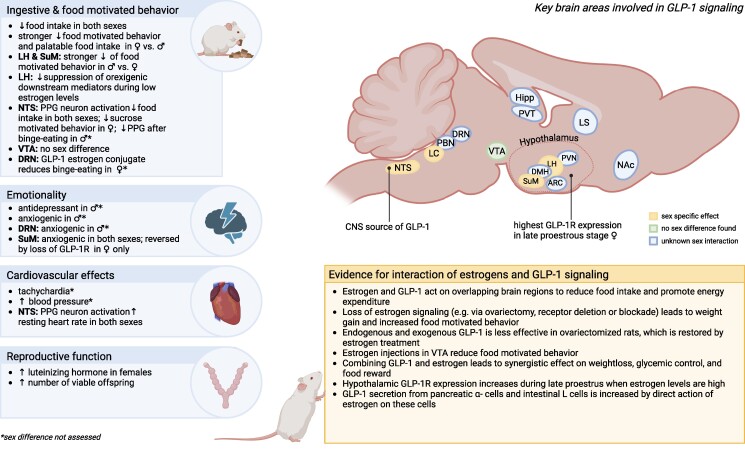
Overview of preclinical evidence for sex-specific effects of GLP-1 and GLP-1R activation and their interaction with estrogens. Created with biorender.com. Abbreviations: ARC, arcuate nucleus; DMH, dorsomedial hypothalamus; DRN, dorsal raphe nucleus; GLP-1, glucagon-like peptide 1; GLP-1R, glucagon-like peptide 1 receptor; Hipp, hippocampus; LC, locus coeruleus; LH, lateral hypothalamus; LS, lateral septum; NAc, nucleus accumbens; NTS, nucleus of the solitary tract; PBN, parabrachial nucleus; PPG, preproglucagon; PVN, paraventricular nucleus; PVT, paraventricular thalamus; SuM, supramammillary nucleus; VTA, ventral tegmental area.

**Figure 2.**
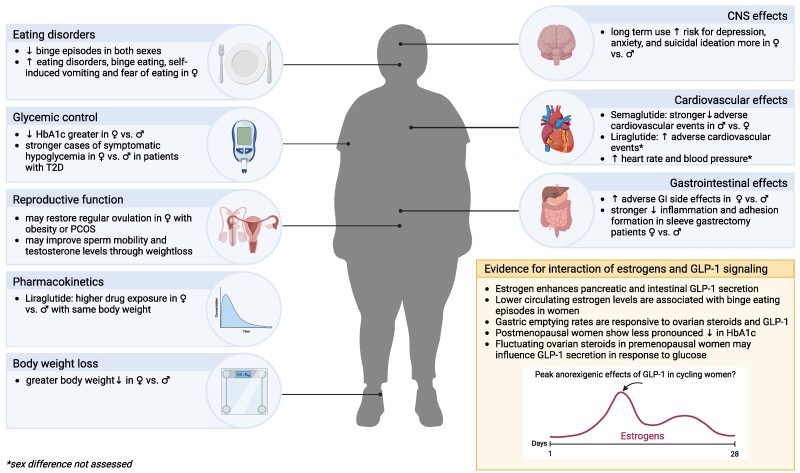
Overview of clinical evidence for sex-specific effects of glucagon-like peptide 1 analogs and their potential interaction with estrogens. Created with biorender.com.

## Sex Differences in Food Intake and Food Motivation Control

The concept of energy balance signals interacting with sex and sex steroids is not new. While relatively few studies ask about sex differences, those that do often find that the physiology and pathophysiology of feeding behavior exhibit sex-specific differences in both animals and humans. While our current focus is on GLP-1 and its analogs, sex differences have been identified for other circulating hormones critical to conveying peripheral energy status to the brain including leptin and insulin (2, 3). Thus the female brain may distinctly integrate peripheral signals to control food intake, with sex steroids playing a pivotal role in these differences (4). A large body of literature supports the idea that sex steroids are key modulators of feeding and metabolism. Gonadal steroids, particularly estrogen, reduce food intake by potentially enhancing the sensitivity of the female brain to other anorexigenic factors such as leptin (4). Estrogen decreases food intake and increases energy expenditure through its action on receptors in the hypothalamus and hindbrain (5-9). In premenopausal women, the ovaries secrete 17β-estradiol as the predominant estrogen in a cyclic manner, whereas in men and postmenopausal women, adipose tissue serves as the primary source of estrogen. Reduction of estrogen levels in female rats via ovariectomy leads to weight gain (10, 11). Similarly, women entering menopause, characterized by reduced estrogen levels, reliably gain weight, underscoring the evolutionary conservation of the relationship between gonadal hormones and body weight (4, 12, 13).

Food intake is also affected by extra-homeostatic areas involved in reward, habits, and decision-making. Reward or motivated behavior is closely linked to the mesocorticolimbic neurocircuitry, particularly the ventral tegmental area (VTA) and its dopaminergic projections to the nucleus accumbens (NAc) (14, 15). Increased dopamine release in the NAc is typically associated with heightened motivated behavior, and palatable food is known to induce dopamine release in the NAc (14). Estrogen receptors are expressed in the VTA, allowing estrogen a direct effect on dopamine neurons, and the dopamine system is highly responsive to sex hormones (16, 17). Female rats also exhibit stronger motivation for palatable food than male rats, yet this motivation is significantly reduced during phases of the cycle when estrogen levels are high (11). Gonadectomy markedly increases food-motivated behavior and food-seeking, even before changes in food intake are observed (11). Estrogens can directly modulate the mesolimbic reward circuitry to decrease food reward behavior (11). While less is known about sex differences in human food reward control, the connectivity in brain regions that regulate reward differs between men and women, suggesting that food reward behavior may be a product of different brain activation patterns across sexes (18).

## GLP-1 and Its Analogs: The Basics

GLP-1 and its receptors have emerged as a successful therapeutic target for the treatment of type 2 diabetes and, more recently, obesity (19, 20). They are also currently considered for multiple additional indications including in the treatment of cardiovascular disease, neurodegenerative disease, and substance use disorders. A variety of GLP-1-based therapeutics have been or are still available on the market including exendin-4, liraglutide, dulaglutide, and semaglutide. Moreover, combination treatments like tirzepatide, which pairs GLP-1 analog with glucose-dependent insulinotropic polypeptide, are emerging and predicted to grow. Overall, GLP-1-based therapies are rapidly gaining popularity: a staggering 1 in 8 US adults aged 18 years or older reports having used a GLP-1 analog medication (21). However, despite their widespread use, some aspects of GLP-1 function, especially pertaining to its role in the central nervous system (CNS), remain unexplored—an important gap considering the increasing number of patients receiving this treatment (22, 23).

GLP-1 affects glucose homeostasis through its concerted action on pancreatic islet hormone secretion, glucose tolerance, gut motility, and appetite. Yet most available evidence points to CNS as a major target for GLP-1 and the analogs' effects on appetite (24, 25). In fact, derived from preproglucagon (PPG), GLP-1 is produced not only in the periphery (intestine, pancreas) but also in the CNS, by neurons located in the nucleus of the solitary tract (NTS) (26). Peripherally released GLP-1 and its stable analogs (eg, exendin-4 or liraglutide) administered peripherally can access the CNS by crossing the blood–brain barrier (BBB) directly (27) or by interacting with the circumventricular organs. This suggests that peripheral, clinically relevant route of administration of GLP-1 analogs, allows them to reach CNS targets. The central application of GLP-1 receptor agonists potently decreases food intake, likely through GLP-1 receptor (GLP-1R)-expressing targets in multiple brain areas including in the hypothalamus and brainstem (24, 26, 28-30), nuclei also targeted by estrogen (31), supporting a potential interaction. Ample evidence also points to a major role of CNS GLP-1R in motivated behavior control—or in clinical terms food reward or cravings—for food but also substances of abuse (32-39). The potent suppressive effect on food-motivated behavior by GLP-1 and its analogs is also driven by multiple distributed brain GLP-1R populations including those in the VTA, NAc, and lateral hypothalamus but also the NTS (32-37).

## Sex Differences in CNS Actions of GLP-1: Preclinical Evidence

While there are significant sex differences in the feeding responses elicited by different CNS GLP-1R populations, the extent and directionality of the uncovered differences depend on the neuroanatomical location of the GLP-1R population. Female rats can exhibit a far more robust GLP-1R activation-induced motivated behavior suppression than male rats, even though central administration of GLP-1R agonists results in a similar ingestive behavior suppression of rat chow in both sexes (40). Moreover, GLP-1R activation suppresses ingestion of palatable food more potently in females (40). However, compared to male rats, a much weaker suppression of food-motivated behavior following pharmacological activation of GLP-1R in the supramammillary nucleus and the lateral hypothalamus was identified (33, 41). Also, selective VTA GLP-1R activation did not produce any sex divergence in feeding or food-motivated behavior, indicating that GLP-1R populations driving the female advantage are located elsewhere. Differential GLP-1R expression or availability in various brain regions might be 1 potential mechanism for the resulting effect divergence. Interestingly, there is some evidence that more stark sex differences in receptor expression emerge in adulthood and might not be found in younger rodents (33, 41).

Another reason for the overall enhanced effect of GLP-1 and its analogs on feeding behavior in females is the ability of these substances to synergistically interact with estrogens. Supporting this idea are multiple preclinical studies indicating that estrogen enhances the effects of GLP-1 primarily on food-motivated behavior but also, to an extent, ingestive behavior (11, 40, 42). Combining GLP-1 with estrogen into a stable conjugate that stimulates all GLP-1R-expressing cells but only those estrogen receptor-expressing cells that contain GLP-1R leads to better glycemic control and synergistic weight loss, as well as a reduction in food reward, the latter 2 mediated by the CNS (42, 43). Moreover, combining GLP-1 with estrogen led to an activation of brain areas that were not affected by either of the substances alone given at doses matching those delivered by the conjugate (42), suggesting that when acting in the presence of estrogen GLP-1 has the potential to have a wider or different effect on the brain. Conversely, removal of gonadal steroids by ovariectomy led to a diminished ingestive behavior response to GLP-1 in female rats and reduced brain activation of some (eg, paraventricular nucleus) but not all (eg, NTS) brain regions (44). Moreover, the satiating efficacy of endogenous GLP-1 is reduced in ovariectomized female rats and is restored by estrogen treatment in these rats (45).

The exogenous addition of estrogen, pharmacological or surgical removal of estrogen signaling (ovariectomy), and gonadal steroids influence GLP-1 or GLP-1 analog's effects on feeding behavior. Gonadal steroids can also influence GLP-1R expression in the brain and other tissues—in the whole hypothalamus, for example, the highest GLP-1R expression is found in the late proestrus stage (46), providing further mechanistic support for potentially higher sensitivity to GLP-1 effects during this phase of the cycle (33, 47). Natural fluctuations of gonadal hormones can also alter brain reception of the GLP-1R activation. For example, in the lateral hypothalamus exendin-4 is less potent at inducing anorexic downstream mediators like neurotensin or IL-6 and at suppressing downstream orexigenic mediators (eg, orexin) when tested in estrous cycle phases in which estrogen levels are low (33, 47). The same study also indicates that, overall, both quantitative and qualitative sex differences exist in the downstream signals engaged by GLP-1R activation. For example, GLP-1R activation suppresses orexins only in females but not in males, while the IL response to GLP-1R activation is much less potent in females compared to males.

Importantly, as many of the aforementioned studies availed direct central drug application, thereby bypassing peripheral metabolism, the resulting sex differences are separable from potential differences in peripheral drug metabolism, BBB crossing, body weight, or fat depot differences—all important factors that add another layer of complexity when interpreting sex differences considered in clinical data.

Even though males have much lower circulating levels of estrogen compared to females, they still do have physiologically relevant levels of this hormone, and even in male brains estrogen receptors are expressed in brain areas known to control feeding and metabolism. Proof of principle for the synergistic interaction of these 2 systems also in males comes from data showing that GLP-1-estrogen conjugate applied peripherally or directly to the CNS has superior effects of body weight loss, fat mass, or food-motivated behavior suppression in males (41, 42). However, even the low levels of estrogen in males may be required for a complete effect of GLP-1R activation on feeding behavior. Intriguingly, a blockade of central estrogen receptors reduces the effect of a GLP-1R analog (exendin-4) on food-motivated behavior in both sexes, indicating that estrogen may modulate the effects of GLP-1 analogs not only in females but also in males (40). The effects of exendin-4 on reward were also diminished by pretreatment with the estrogen receptor-α-selective antagonist, suggesting that signaling via the estrogen receptor-α specifically is necessary for GLP-1R analog's reward-reducing effects (40).

Both the potentiation of GLP-1 effects on body weight by estrogen, as well as the more robust food motivated behavior suppression achieved by GLP-1R activation in females may translate to higher efficacy of GLP-1 analogs in patients. In a real-world situation, suppression of both ingestive (eating food already available) as well as motivated behavior (willingness to put in effort to obtain food) is required for effective reduction in food intake and therefore also weight loss, and based on results from animal models GLP-1R agonists can suppress both, and more potently in females. This is indeed supported by clinical data discussed below, where women show a more pronounced weight loss after the same dose of GLP-1 analogs.

GLP-1 has also been shown to interact with androgens. In mouse and human islets, the insulinotropic effect of dihydrotestosterone depends on GLP-1R activation, and dihydrotestosterone amplifies the incretin effect of GLP-1 (48). Testosterone exerts genomic and nongenomic actions to enhance GLP-1-stimulated insulin exocytosis in male β cells (49). Dihydrotestosterone was also shown to increase GLP-1R expression in mouse pancreas (50).

While all the thus far discussed studies examined the effects of GLP-1 signal reception (by exogenous GLP-1 or analog application), very few studies assessed the effects of potential endogenous GLP-1 release in both sexes. Both optogenetic and chemogenetic GLP-1-producing PPG neuron activation in rats leads to a robust ingestive behavior suppression, with a similar effect size between males and females (51). These strategies also reduced sucrose-motivated behavior in female rats (51). However, neither optogenetic nor chemogenetic PPG neuron activation reduced motivated behavior for sucrose in males (51). Therefore, there is a sex difference in the ability of GLP-1-producing neuron activation to control motivated behavior for food as well as palatable food intake. In line with a lack of sex differences in chow intake after PPG neuron activation, no sex differences were observed in glucose control in mice after ablation of PPG neurons (52).

**Figure 3.**
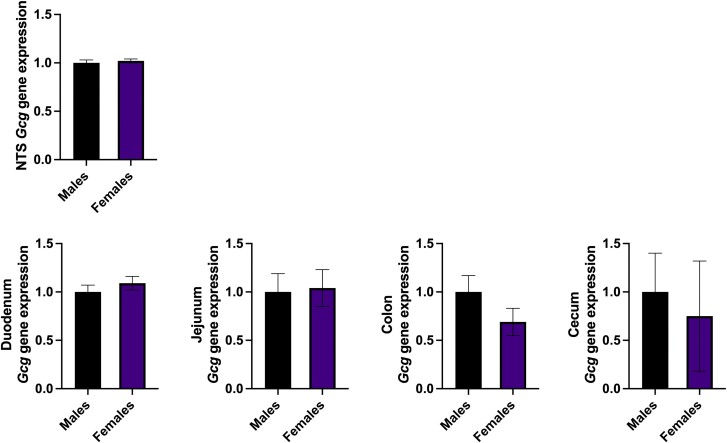
The expression of *Gcg*, the glucagon-like peptide 1 precursor gene, is similar in adult male and female rats in the brain's nucleus of the solitary tract as well as throughout the gastrointestinal tract. Tissue was obtained from adult male (n = 3-14) and female (n = 3-9) Sprague–Dawley rats. Gene expression was determined by RT-PCR, as described previously (33). Unpublished observations.

There is also a paucity of data allowing comparison of the precursor gene (*Gcg*) expression in either the brain or the gastrointestinal (GI) tract. However, brain *Gcg* expression in rats was shown not to be different between the sexes (40). The effect of estrous cycle on this expression remains unknown. Here we confirm these results and further show that *Gcg* expression is also similar between males and females at all levels of the GI tract (Fig 3; unpublished observations). While this still leaves the possibility that posttranscriptional steps differ between the sexes, or that GLP-1's release mechanisms differ, at least at the level of precursor gene expression, no differences were found in rats. There is, however, recent evidence from mice and humans that GLP-1 secretion from pancreatic α- cells and intestinal L cells is increased by the direct action of estrogen on these cells (53). These results are further supported by clinical studies, showing higher GLP-1 plasma levels in women in response to an array of nutrients (discussed in the next section).

## Sex Differences in Clinical Results

### Body Weight and Glycemic Control

Women consistently show more pronounced weight loss with all GLP-1 analogs, including exendin, liraglutide, dulaglutide, and semaglutide (54-57), which indicates a specific response of GLP-1R activation rather than a GLP-1 analog-dependent sex divergence. This is reflected by women achieving greater mean weight reduction in the STEP 1, 2, and 4 trials, conducted in overweight or obese patients with or without diabetes (58): 14.0% vs 8.0% in STEP 1, 7.5% vs 4.6% in STEP 2, and 16.2% vs 9.3% in STEP 4 in women and men, respectively (58-62).

In patients with obesity undergoing sleeve gastrectomy, liraglutide has been explored for preoperative management, showing more pronounced effects in females by reducing inflammation and adhesion formation (63). On the other hand, semaglutide significantly reduced major adverse cardiovascular events in patients with obesity, with a more pronounced cardiovascular benefit in men (64).

Sex divergence in blood glucose control exerted by GLP-1 analogs, as indicated by hemoglobin A1c improvements, is less consistent than what was found for body weight effects of these drugs, although more studies report a more pronounced response in females (54-57, 65-68) as compared to those finding a higher percentage of males reaching target hemoglobin A1c (55) or no sex difference (56, 69, 70). Intriguingly, and consistent with the role of gonadal steroids in the enhanced responses in females discussed in the preclinical data section, 1 study found that older postmenopausal women do not show the pronounced response found in younger women (68). This finding adds a layer of complexity to how sex differences are considered and indicates that comparisons should ideally be stratified by pre- vs postmenopausal age, to assess the extent of activational vs organizational contribution to the potentially detected sex difference. It also indicates that analyzing mixed-age groups may reduce the chances of detecting potential differences, if such for example exist exclusively in premenopausal women. In line with these observations, estrogen has been shown to enhance antidiabetic actions of GLP-1. There is preclinical evidence indicating that estrogen combined with GLP-1 is superior to GLP-1 alone in the protection of diabetic male mice against carbohydrate-induced beta cell failure (71). Moreover, estrogen enhances GLP-1-mediated protection of insulin-deficient diabetes in male mice (72). It also amplifies antiapoptotic pathways activated by GLP-1 in male human β cells (72).

Part of the improvement in body weight and blood glucose levels has been ascribed to the GLP-1 analog’s effect on gastric emptying (73). Gastric emptying rates differ between sexes and tend to be slower in women; they are also responsive to ovarian steroids (74). Yet, sex differences in the gastric emptying impact of GLP-1 analogs have not been consistently analyzed.

Endogenous intestinal or pancreatic GLP-1 responses might also differ between the sexes. This might be due to higher circulating levels of estrogen in women since estrogen was shown to increase total GLP-1 secretion from human pancreatic alpha cells and intestinal L-cells (53). Naturally fluctuating gonadal steroids in premenopausal women might also contribute to GLP-1 secretion in response to glucose as women in the luteal vs follicular phase showed a markedly greater GLP-1 response to a 50 g oral glucose drink (75). Also, monosaccharide and fiber preload increased and extended the GLP-1 release by breakfast in women but not in men (76). Moreover, premenopausal women have a markedly greater GLP-1 response to intraduodenal glucose than men, with the 60-minute area under the curve 2.5-fold higher in women (77). Interestingly, glucose-dependent insulinotropic polypeptide levels were not sex divergent in the same study, suggesting that among incretins GLP-1 may be more prone to sex differences, at least in the setting tested. Overall, these data suggest that endogenous GLP-1 responses to physiological stimuli may differ between the sexes in humans.

### Adverse Effects

Some studies suggest that women tend to discontinue GLP-1R agonist treatment more than men. In 1 study, nearly 50% more women discontinued the treatment compared to men, though this difference did not reach significance in this study, which was not powered for sex difference detection (78). Furthermore, twice as many women compared to men discontinue GLP-1R agonist treatment due to GI side effects (trend *P* = .07). Indeed, adverse GI effects such as nausea may be more frequently experienced by women as demonstrated by a study on cotadutide (dual agonist of the GLP-1 and glucagon receptors), which attributed this effect to slower drug absorption rates in women (79). For cotadutide, however, the glucagon component might also contribute to nausea.

In a trial testing liraglutide in patients with type 2 diabetes, 28% more women than men reported adverse effects of the treatment; in another trial testing dulaglutide, 41% more women reported adverse events (80). Although other trials, for example the SUSTAIN 6 trial evaluating semaglutide for potential cardiovascular benefits in patients with type 2 diabetes, did not find a sex difference in the rate of reported GI events (51% more than placebo in men, 47% more than placebo in women) or in the rate of treatment discontinuation due to adverse events (76% more than placebo in men, 127% more than placebo in women). Interestingly, more women than men reported symptomatic hypoglycemia in this trial (0% over placebo in men and 13% above placebo in women) (81).

For clinical studies, it would be important to consider these data as a function of potentially higher dose application because of body weight or pharmacokinetics differences between the sexes. Unfortunately, it is difficult to assess potential sex differences in pharmacokinetics and tolerability of, for example, semaglutide, as a large fraction of the available data was either tested exclusively in male human, primate, or rat subjects (82) or not presented disaggregated by sex (83, 84). In the latter safety study, in line with most drug safety studies, in the healthy control group women comprised only 35% of the subjects, tilting potential conclusion to the male response, if different from that in females. Sex-disaggregated data are, however, available for liraglutide, where a 32% higher drug exposure was found in females than males with the same body weight (85). Intriguingly, even with similar drug exposures, women have been reported to experience more undesired effects of the semaglutide treatment compared to men (86). That the lower, on average, body weight of women might not be the principal factor explaining the higher efficacy of the drugs in women is suggested by data indicating that women who weighed more than men on average lost more weight after liraglutide treatment (87).

## CNS Effects of GLP-1 Analogs Outside of Metabolism

### Depression

Assessing GLP-1 analog impact on mental health in patients with diabetes or obesity is complicated by the potential influence of these metabolic disorders on mental health. Some, but not all, studies indicate a positive correlation between metabolic syndrome and anxiety disorders or depression (88-92). Since improvements in mental health are often noted upon significant weight loss, associated with improvements in inflammatory and metabolic parameters, this may suggest that GLP-1 analogs are generally expected to improve mental health simply as a byproduct of their metabolic effects. However, given that GLP-1Rs are located in multiple brain areas tightly associated with emotionality control, including the dorsal raphe serotonin neurons, as well as the hippocampus, it is reasonable to also expect that GLP-1 analogs have the potential to directly alter emotionality (93, 94). In line with this expectation, GLP-1R agonists, including exendin-4, have been shown to exhibit antidepressant properties, independently of weight loss, in preclinical studies (95-97). Only males, however, were evaluated in these studies, leaving the potential impact of the drugs on depression-like behavior in rodent models on females unknown. Clinical studies probing whether GLP-1 analogs alter the incidence of depression produced mixed results. While some indicate an improvement in parameters associated with depression (98), others show an increased risk, especially after longer periods of drug exposure and with the newer drugs with a longer half-life (99, 100), though some other studies indicate a higher risk with liraglutide compared to semaglutide (which in that particular study was associated with reduced risk for psychiatric events) (101).

Some of these studies also suggest that sex does matter when it comes to the potential interaction between GLP-1 analogs and depression (99). A recent large community-based cohort study including over 160 000 patients in each group found that women show a hazard ratio of 3.16, compared to males at 2.89, of being diagnosed with depression after GLP-1R analog therapy. An even larger sex effect was found for suicidal ideation or attempts (2.53 in women vs 1.70 in men) (99). Interestingly, in the study noting a benefit of semaglutide over other antiobesity medications on suicidal ideations, women appear to derive slightly less benefit than men (98). Another important factor to consider is that the association between obesity and suicide might be greater for women than men (102).

Overall, it appears that sex might be an important factor when assessing risks or benefits from GLP-1 analogs. It adds to other variables recently suggested to affect the interaction of GLP-1R agonists with depression, which include existing and treated diabetes or obesity, age, duration of treatment, whether comparisons are made to control groups receiving placebo or other antiobesity medications, and whether patients with preexisting psychiatric diagnosis are included. Given how nuanced the impact of GLP-1 analog treatment on depression might be, it is crucial to consider sex as a vital variable and report it and analyze it as such in preclinical as well as clinical studies on this association.

### Anxiety

Women are significantly more prone to mood and anxiety disorders compared to men, with studies showing that anxiety disorders are twice as prevalent in women vs men in the United States (103). Additionally, meta-analyses indicate that the association between obesity and anxiety disorders may be more pronounced in women than in men (89, 104-106). Despite their potential antidepression effects, preclinical studies overwhelmingly indicate that acute treatment with GLP-1 analogs is anxiogenic (95, 107-109). Both GLP-1 and its long-lasting analog, exendin-4, can induce anxiety-like behavior in male rodents when administered acutely (95). These effects were observed in various tests such as the black and white box, elevated plus maze, and open field test when the drug was administered acutely via intraperitoneal injection into the lateral ventricle or directly into the dorsal raphe nucleus (95, 108-110). This anxiogenic role of GLP-1 is likely tightly linked with the role brain GLP-1Rs play in interoceptive stress responses (108, 111). Supporting this idea are data showing that GLP-1 increases levels of the stress-activated hormones ACTH and corticosterone when administered directly into the rat brain, at least in male rats (108). Moreover, the anatomy of the PPG-neuron projections is ideally situated to provide interoceptive information from the hindbrain to the limbic areas mediating the responses to stress (111, 112).

Unfortunately, few preclinical studies evaluate both sexes in this context. However, 1 report indicates that both male and female rats show acute anxiogenic responses to GLP-1R activation, at least when the drug is delivered specifically to the supramammillary nucleus (113). Interestingly, improvements in anxiety-like behavior after loss of GLP-1R were only noted in females, potentially suggesting females rely more on this system to maintain a baseline of normal anxiety-like behavior (113). Some clinical studies also support an increased incidence of anxiety diagnosis after GLP-1 analogs, with a more pronounced impact on women (hazard ratio of 2.19 vs 1.89 in women and men, respectively) (99), although more research evaluating this question in preclinical and clinical settings is needed for a more robust conclusion.

#### Eating Disorders

Eating disorders such as binge eating disorder (BED) and anorexia nervosa (AN), both of which are significantly more prevalent in women, exhibit distinct patterns of GLP-1 regulation, underscoring the hormone's complex involvement in disordered eating.

BED involves binge episodes characterized by the intake of large quantities of palatable, calorie-dense foods in a relatively short amount of time (114) and is associated with elevated fasting GLP-1 levels compared to non-binge-eating individuals. For example, intermittent access to high-fat foods raises plasma GLP-1 but suppresses PPG in the NTS in male rats (115). This interesting dichotomy suggests that while systemic GLP-1 increases may be an adaptive response to control intake, central GLP-1 signaling reduction might counteract the peripheral changes and disinhibit reward-driven intake, potentially enhancing susceptibility to binge behaviors (116). However, as only male rats were tested in the aforementioned study, and since PPG neuron activation was recently shown to have divergent effects on food-motivated behavior and palatable food intake in male vs female rats (51), it is also likely that this PPG neuron accommodation disinhibits female behavior more, contributing to the higher prevalence of binge-like behavior in females, a speculation that needs to be tested. In women, however, GLP-1 levels in response to a meal showed no differences between obese subjects with or without BED (117). Circulating estrogen levels are inversely associated with binge eating in women, and removal of ovarian hormones increases binge eating (118-120). In female mice, while GLP-1 delivered to the dorsal raphe nucleus reduced binge-like eating, the effect was much more pronounced with GLP-1–estrogen conjugate (121). Early open-label studies with relatively small sample sizes using GLP-1R agonists such as liraglutide (122, 123) and dulaglutide (124) show promising effects in reducing binge episodes and weight in BED, but further research is needed to confirm their benefits across diverse populations. Here, some studies did include both sexes but failed to test for sex-specific effects. In 1 study, repeated measures analyses of covariance were performed to adjust for sex, but the effect of sex was not reported (122). Similarly, another study included both sexes but only mentioned a preliminary correction of body composition for age, sex, and body mass index without specifying how this correction was performed (124), while another did not mention the sex of participants at all (123). For an extensive review of the emerging role of the GLP-1 system in BED, see (116).

AN is characterized by restrictive intake patterns and is associated with lower baseline GLP-1 levels compared to healthy individuals. This may be an adaptive reduction—an attempt to restore energy balance and support increased appetite by diminishing satiety signals, aiding in refeeding efforts (125). However, as treatment progresses and GLP-1 levels rise during refeeding, they may remain elevated, potentially contributing to delayed gastric emptying and lower postprandial glucose levels—factors that can complicate the refeeding process as the body adjusts to normal intake (126). This potentially suggests that pharmacological blockade of the GLP-1R might be a promising target for AN patients, at least during later stages of treatment. Likely due to the higher prevalence of AN among them, these 2 studies focused exclusively on adolescent girls, thereby limiting insights to female biology at a specific developmental stage. This narrow focus, however, also restricts our understanding of potential sex differences in GLP-1's role in AN pathology, as it overlooks biological and neurodevelopmental factors that may present differently in males and across age groups.

Together these findings highlight GLP-1's nuanced role in eating disorders, with potentially maladaptive effects of GLP-1 in some aspects of BED and AN. In BED, GLP-1 dysregulation in the brain may contribute to compulsive intake, while in AN, reduced GLP-1 initially supports refeeding, though elevated levels during recovery may reintroduce strong satiety signals. While GLP-1R agonists show promise for managing binge eating, there is concern about their misuse in populations with eating disorders, where their appetite-suppressing effects could exacerbate restrictive eating behaviors. This is underscored by a recent report on psychiatric adverse events associated with GLP-1 analogs, which found an increased odds ratio for eating disorders, binge eating, self-induced vomiting, and fear of eating among those using GLP-1 analogs (127). Importantly, women were more affected by psychiatric adverse events compared to men.

### Cardiovascular Effects

GLP-1 analogs are gaining interest for their cardiovascular effects beyond glucose or body weight control, as both central and peripheral activation of GLP-1 receptors has a range of impacts on heart rate (HR) and blood pressure (28, 128). Interestingly, increased resting HR is a potential adverse effect included in the patient leaflet for GLP-1 analogs (eg, semaglutide), and some physicians express concern over the increases in HR seen in patients. Some studies report increases of 2 to 3 beats per minute (bpm), yet others, including a Holter electrocardiogram study, found mean increases of 13 bpm (129). The sex of the patients was not provided; however, considering well-established sex differences in resting HR, including sex as a variable would have been of value here, and its absence represents a missed opportunity.

Systemic and intracerebroventricular administration of exendin-4 or even native GLP-1 in male rats increases HRs up to 600 bpm (nearly double rat resting HR) and blood pressure for several hours postadministration (28, 130, 131). GLP-1 analogs likely influence cardiovascular function by sympathetic and parasympathetic pathways, at least in male mice (132). In male rats, a tachycardic response can be elicited by GLP-1R activation in the brainstem (28, 133). Research in decerebrate male rat models further supports the role of the caudal brainstem in GLP-1-mediated cardiovascular responses, where hindbrain-restricted GLP-1R activation was sufficient to suppress food intake while inducing tachycardia, independent of hypothalamic or forebrain input (28).

In addition to the robust tachycardic responses obtained with exogenous GLP-1R activation, endogenous GLP-1 contributes to resting HR regulation, as GLP-1R knockout (male) mice exhibit lower resting HRs (134). Furthermore, chemogenetic activation of NTS PPG neurons suggests that central GLP-1 release can increase HR (128). Interestingly, these neurons do not contribute to tonic cardiovascular regulation; thus their role may be limited to responses to acute stress rather than baseline control of cardiovascular function. While this latter study was conducted in mixed-sex cohorts, the authors acknowledge that the study was not designed or powered to assess sex differences, limiting the ability to determine potential sex differences in these cardiovascular effects.

These preclinical findings highlight GLP-1's broad involvement in cardiovascular regulation, especially through brainstem and autonomic mechanisms. The potential sex effects, however, remain unknown, as most of the studies excluded females, and in cases that did include females, sex-disaggregated data were not shown. In clinical studies treatment with liraglutide was associated with an increase in HR (mean difference 7 bpm) and more serious cardiac adverse events (3% vs 10% for placebo and liraglutide, respectively) in chronic heart failure patients with and without type 2 diabetes (135). Unfortunately, females represented only 12% of the patients, and sex-disaggregated data were not provided, leaving any potential sex interaction unknown (135).

Other clinical data, however, also indicate that longer-term use of GLP-1 analogs is associated with cardiovascular benefits (136), possibly with a more pronounced cardiovascular benefit in men (64). Semaglutide reduces major adverse cardiovascular events, heart failure composite, cardiovascular death, and all-cause death in patients with atherosclerotic cardiovascular disease and overweight or obesity and heart failure. A majority of the patients in this study were male (73%). While the study concluded that the clinical benefit was independent of sex, it does appear that some of the benefits were more pronounced or more consistent in males: major adverse cardiovascular event HR 0.71 (0.58-0.88) in males vs 0.78 (0.52-1.19) for females; the interaction was not significant. The mean age in this study was 61, indicating that likely most of the women included were postmenopausal, which, based on the interactions with estrogen discussed earlier, could explain the lower and more variable benefits in women.

## Brain Access

The profound impact of GLP-1 and its analogs on the human brain activity is unquestionable (137-139); to what extent it is direct or indirect is still debated. Endogenously released intestinal GLP-1, given its very short half-life, is unlikely to reach brain GLP-1R beyond circumventricular areas. Thus, the widely distributed brain GLP-1R populations are likely to primarily respond to the endogenous GLP-1 delivered by the PPG neurons. The synthetic GLP-1 analogs currently on the market may reach both peripheral and central GLP-1R populations. It is clear that the blockbuster antiobesity therapeutics do access the brain to exert much of their anorexic action; the extent of this brain penetration, however, is under debate and has been suggested to cover only the hypothalamus and hindbrain (140) or extend to many other populations including those found deep in the brain, for example in the hippocampus or NAc (27, 141, 142). It has also been suggested that brain penetrance differs between different GLP-1 analogs, with nonacylated and non-PEGylated analogs like exendin-4 or lixisenatide showing significant rates of brain influx, while liraglutide or semaglutide do not have reliable BBB crossing (143), at least in male mice. Since this topic has been extensively reviewed recently (144), here we focus on the potential sex differences in this process and inclusion (or lack thereof) of females in key studies. Most of these studies were conducted exclusively in male rodents; even the newest study showing semaglutide distribution near circumventricular organs or brain regions near the ventricle (eg, lateral septum) only examined male mice (145). However, we identified 1 study using female mice, which found a clear presence of 2 different GLP-1 analogs, liraglutide and lixisenatide, in the brain after peripheral injections, indicating that they also enter the brain in females (142). Given that other studies did not find liraglutide in male rodent brain, it is tempting to speculate that this might hint at a potential sex difference, but at this point considering that different methods to study brain penetration were used in these studies, a direct comparison is likely not warranted, leaving this question wide open. Moreover, 1 recent study assessed the presence of peripherally injected semaglutide in the NAc of male and female rats and found the GLP-1 analog in the NAc of both males and females (141). Still, studies examining systematically whether the potential brain penetration of different GLP-1 analogs exhibits sex divergence are desperately needed to be able to determine whether brain entry of these clinically utilized drugs differs between the sexes. It will be especially important to test brain penetrance in both sexes under conditions of obesity, diabetes, or any other diseases that are indicated for GLP-1 therapeutics and have the potential to alter the BBB. This knowledge might be key to understanding some of the emerging sex-interacting therapeutic and side-effects profiles of these medications.

## Effects on Reproductive Function and Fetal Development

An increasing number of women of reproductive age are diagnosed with type 2 diabetes and obesity, and many now turn to GLP-1 analogs for the treatment of both conditions. According to current product labels, use of the GLP-1-based therapeutics should be discontinued 2 months before conception, as well as during pregnancy and lactation. Yet scientific and media reports indicate that women who have been struggling with infertility (eg, those with polycystic ovary syndrome or obesity but also women on oral contraceptives taking GLP-1 analogs) may experience an increased rate of unplanned pregnancies. It has been suggested that GLP-1R-targeting drugs, especially tirzepatide, significantly reduce the absorption of oral contraceptives (146). Semaglutide and liraglutide were, however, shown not to alter the bioavailability of oral contraceptives, at least in postmenopausal women (147, 148). While currently available clinical evidence does not support altered contraceptive bioavailability at least by single GLP-1R agonists (149), it is possible that female fertility is affected in other ways by GLP-1 analogs directly or indirectly. For example, for women with polycystic ovary syndrome or obesity struggling with infertility, the weight loss triggered by GLP-1 drugs might restore regular ovulation. A direct effect of GLP-1 and its analogs on fertility is supported by the expression of GLP-1R in ovaries, the pituitary, and the hypothalamus, potentially allowing the drug or native peptide to control reproductive axis at all levels (46). Moreover, dosing rats with GLP-1 stimulates the production of LH in female rats, which triggers ovulation in rats and humans (46). Rats given GLP-1 also had increased numbers of viable offspring compared to controls (46). Moreover, brain GLP-1 administration to prepubertal female rats promotes puberty onset (46). While fewer studies asked about the interaction of GLP-1-based therapeutics and reproductive axis in men, there is some evidence suggesting improvements in reproductive functions at least in the context of improving obesity. For example, in obese men with functional hypogonadism, liraglutide improved reproductive function and was associated with a significant increase of LH and FSH (150). In obese and diabetic men, semaglutide was recently shown to improve sperm morphology, total testosterone levels, and symptoms of hypogonadism (151). In both studies, however, it is likely that marked improvements in obesity and diabetes induced by liraglutide or semaglutide were the primary drivers of the improved reproductive function. Moreover, both studies included a relatively small sample (25-30 men). Thus, the current results need to be replicated in a larger sample, and more studies are needed to determine whether endogenous or exogenous GLP-1 or analogs can affect the reproductive axis directly in men.

The increased number of women of reproductive age taking GLP-1-based therapeutics, along with the potentially unexpected restart of fertility, might indicate that an increasing number of infants will be exposed to GLP-1 agonists prenatally; the consequences of this exposure remain largely unknown. A review of 39 human and animal studies on the topic, however, reached a concerning conclusion that GLP-1 agonists reduce fetal survival, weight, growth, and skeletal ossification and can induce major congenital anomalies (152). Yet a more recent study indicates that this class of medications does not increase major congenital malformations in women with type 2 diabetes (153). Together this clearly indicates that the issue is far from resolved and, considering the potential human life cost, needs urgent attention. Due to the high molecular weights of GLP-1 analogs, mother-to-fetus placental transfer and therefore fetal exposure may be very limited (152). In a case report where a woman carried a pregnancy to term while taking liraglutide, drug levels in umbilical vein blood were below detection levels (154). However, GLP-1 analogs have also been found in the breastmilk of animals and humans (152, 155), but in the latter study the relative infant dose of semaglutide was only 1.26%, significantly lower than the standard safety threshold of 10%. Nonetheless, more studies are needed to assess the effects of GLP-1 analogs on (neuro-) development to exclude adverse effects.

A large body of literature also indicates the potential for therapeutic exploitation of the GLP-1 system in treatments of substance use and neurodegenerative disorders (156-159). Given the sex differences detected in preclinical models of GLP-1 and the analogs’ action on motivated behavior described earlier and very well-established sex differences in various aspects of substance use, as well as well-established sex differences in presentation and prevalence of some neurodegenerative disorders, it is not unlikely that considerable sex differences also exist in endogenous or exogenous effects of GLP-1R receptor activation in both domains. However, given the paucity of literature examining sex differences in the effects of GLP-1 analogs on these domains, along with space constraints of the current review, these topics, while very important, are not covered here.

We also note that statements of sex difference or the lack thereof from rodent studies that specified that both sexes were used but did not give the number of subjects per sex or show disaggregated sex data were largely not considered in this review.

## Conclusion

Careful investigation of sex differences has the potential to not only fill in the gap for missing research or information for female subjects, which are still often omitted from preclinical studies, but also to more appropriately adjust the doses for both sexes to, for example, increase the effectiveness for body weight loss in men and for women to reduce side effects. Overall, considering the reviewed-here available animal (Fig. 1) and human (Fig. 2) studies, we did not find evidence for large qualitative sex differences in the therapeutic effect of clinically approved GLP-1 analogs. Men and women and female and male rodents lose weight and eat less in response to GLP-1 and its analogs. However, a growing body of literature highlights quantitative sex differences in how males and females respond to GLP-1 and its analogs. Moreover, there is also evidence for qualitative sex differences in the downstream mediators GLP-1R agonists engage; thus while both males and females might reach a similar in direction behavioral or physiological outcome, they might engage partly different neural signals and brain regions to get there. Caution is also warranted when dismissing smaller quantitative sex differences in the therapeutic or adverse effect outcomes, especially considering the staggeringly large numbers of patients taking GLP-1-based therapeutics. At that scale, small differences can translate to large numbers of patients affected.

Sex-specific dosing remains a controversial topic (160). In the case of zolpidem, a sleep medication that is now Food and Drug Administration recommended at different doses for men and women, studies found clear sex disparities in clearance rate, but many of the sex differences can be explained by body weight differences between the sexes, leaving some to question whether sex differences were the right focus. However, given the large and growing body of preclinical literature highlighting mechanisms outside of body weight contributing to sex differences in effects of GLP-1R agonism (eg, interactions with estrogen), the case for meaningful and potentially clinically relevant differences remains, if not strong, then at least certainly warranting careful attention to sex as an important variable in GLP-1 system research.

Yet, there is still much that remains to be explored. Here we elected to underscore some of the many instances where sex was either not provided, not disaggregated, or not appropriately analyzed to highlight the many missed opportunities to understand sex differences in this system. What also clearly emerges is the paucity of data in female animal models or women in very basic aspects of the science of GLP-1, including in brain penetrance, pharmacokinetics, or the preclinical cardiovascular space.

## Data Availability

Some or all datasets generated during and/or analyzed during the current study are not publicly available but are available from the corresponding author on reasonable request.
